# Critical heat flux enhancement in microgravity conditions coupling microstructured surfaces and electrostatic field

**DOI:** 10.1038/s41526-021-00167-3

**Published:** 2021-10-08

**Authors:** Alekos Ioannis Garivalis, Giacomo Manfredini, Giacomo Saccone, Paolo Di Marco, Artyom Kossolapov, Matteo Bucci

**Affiliations:** 1 DESTEC, University of Pisa, 56122 Pisa, Italy; 2 Department of Nuclear Science and Engineering, Massachusetts Institute of Technology, Cambridge, MA 02139 USA

**Keywords:** Mechanical engineering, Applied physics

## Abstract

We run pool boiling experiments with a dielectric fluid (FC-72) on Earth and on board an ESA parabolic flight aircraft able to cancel the effects of gravity, testing both highly wetting microstructured surfaces and plain surfaces and applying an external electric field that creates gravity-mimicking body forces. Our results reveal that microstructured surfaces, known to enhance the critical heat flux on Earth, are also useful in microgravity. An enhancement of the microgravity critical heat flux on a plain surface can also be obtained using the electric field. However, the best boiling performance is achieved when these techniques are used together. The effects created by microstructured surfaces and electric fields are synergistic. They enhance the critical heat flux in microgravity conditions up to 257 kW/m^2^, which is even higher than the value measured on Earth on a plain surface (i.e., 168 kW/m^2^). These results demonstrate the potential of this synergistic approach toward very compact and efficient two-phase heat transfer systems for microgravity applications.

## Introduction

Pool boiling is an effective means to remove heat: due to the latent heat involved in the evaporation and the fluid motion induced by bubbles, heat transfer coefficients are even two order of magnitude higher than single-phase heat transfer^1^. Critical heat flux (CHF) is the maximum heat flux that can be removed by nucleate boiling heat transfer. At higher heat fluxes, a stable vapor layer blankets the heated surface. This heat transfer regime is known as film boiling. It entails a drastic reduction of the heat transfer coefficient and a consequent and potentially destructive increase of the system temperature. Thus, CHF is a crucial operational limit in many boiling heat transfer applications^1^, as diverse as electronic cooling^2^, nuclear reactor cooling^3^, and thermal management of space stations and satellites^4^. Space stations and satellites are rather special applications, as they operate in microgravity conditions, which affects the dynamics of nucleate boiling and the CHF limit.

Consider the simple case of a horizontal boiling surface. In normal gravity conditions (e.g., on Earth), bubbles arise from the heated surface and, for sufficiently high heat fluxes, coalesce into vapor slugs and columns. However, buoyancy drives the vapor upward and away from the surface^5^. Instead, in microgravity conditions, bubbles barely move. Typically, a large bubble hovers at a short distance from the surface, while many smaller bubbles nucleate and grow underneath. These small bubbles feed the large one, which acts as a vapor sink. The large bubble detaches periodically from the heated surface but hardly moves away due to the lack of gravity. Straub proposed that there are two types of heat transfer mechanisms involved in boiling heat transfer^6,7^. Primary mechanisms are near-wall and include the evaporation at the liquid–vapor–solid contact line or the microlayer evaporation. Such mechanisms are dominated by capillary forces and should not be influenced by gravity. Secondary mechanisms instead transfer heat and mass from the wall to the bulk. They include bubble departure and condensation, and convection induced by bubble motion. All these secondary mechanisms are strongly influenced by buoyancy and cause a boiling performance impairment at reduced gravity levels. In fact, even if a stable boiling process can be obtained in microgravity^8^, CHF encounters a drastic reduction compared to normal gravity conditions. Therefore, enhancing the CHF limit in microgravity conditions is key to the design and operation of more efficient and compact heating and cooling devices for space applications.

Over the past decades, many techniques have been proposed to enhance CHF, mostly in normal gravity conditions. They can be divided into passive techniques, which do not require energy to work (e.g., surface modifications or use of nanofluids), and active techniques, which instead require an energy source (e.g., electric, magnetic, and acoustic fields). On the one hand, significant CHF enhancements (in normal gravity conditions) have been obtained using microstructured surfaces, such as micropillars^9^. The actual enhancement mechanism activated by these micropillars is still debated^10–13^. However, there is a consensus that capillary forces play a critical role, as these surfaces have enhanced wetting properties. On the other hand, an electric field can remove vapor from the surface and break large vapor patches formed by bubble coalescence^14^. The electric field effects (due to dielectrophoresis and electrostriction forces) are particularly pronounced on bubble growth and detachment^15^, bubbles trajectories^16,17^, and vapor–liquid interfaces^18^.

We hypothesize that the simultaneous and synergistic use of these two techniques can significantly enhance the CHF in microgravity conditions. Micropillars should enhance the primary boiling mechanism, as they increase the near-wall capillary forces. At the same time, the electric field should move small bubbles away from the heated surface and avoid the formation of a large hovering bubble, i.e., it enhances the secondary boiling mechanisms.

## Results

### Experimental setup

**Fig. 1:**
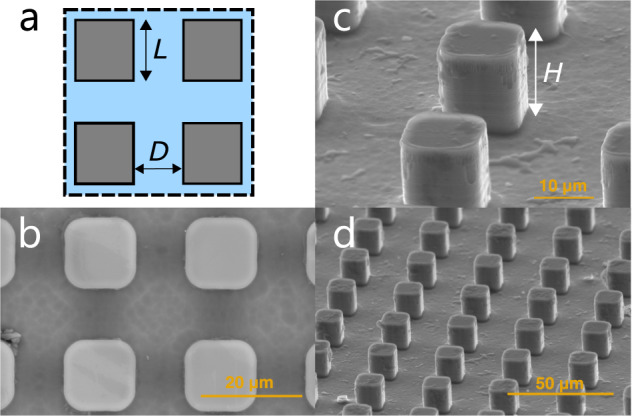
SEM images of the microstructures. **a** Scheme of the square pillars distribution and definition of the pillar side length *L* and spacing *D*. **b** Perspective view in detail of some real pillars at Scanning Electron Microscope (SEM) and definition of the pillar height *H*. **c** Top view of some pillars at SEM. **d** Perspective view of some pillars at SEM.

**Fig. 2:**
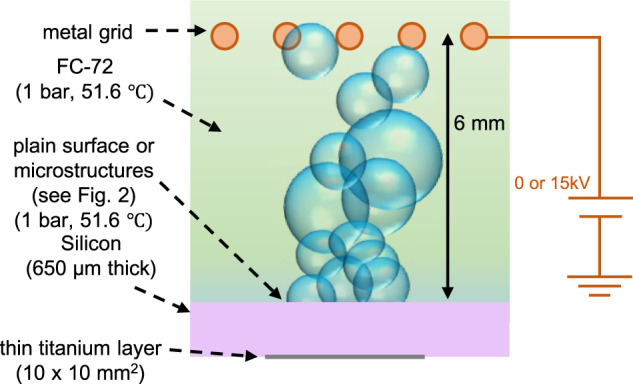
Sketch of the boiling experiment. The conceptual outline of the experiment, consisting of pool boiling in FC-72 fluid that takes place on a heated silicon substrate within an electric field produced by the metal grid.

To demonstrate the potentiality of the enhancement techniques described above, we run pool boiling experiments in normal gravity and microgravity conditions, using plain and microengineered (pillars, see Fig. 1) surfaces and probing the effect of an external electric field. Precisely, we run experiments in the laboratory to collect Earth’s (normal) gravity reference data and during a parabolic flight experimental campaign held by ESA on an Airbus A310-ZeroG (with a maximum measured gravity value of approximately 10^−2^ g). Tests are conducted on plain and microstructured silicon substrates, using degassed FC-72 (a commercial version of n-perfluorohexane whose saturation temperature at 1 bar is 56.6 °C and often used to cool electronics) kept at 1 bar and 51.6 °C (see Fig. 2). The liquid is 5 °C subcooled to avoid excessive vapor accumulation in microgravity.

The boiling process is recorded by a high-speed black and white camera from the side and the temperature of the silicon substrate is acquired via a PT100. The surface is Joule heated by an electrically conductive titanium layer coated on the dry side of the substrate. The areas of the heating layer on the dry side and the structured surface on the boiling side are both 1 × 1 cm^2^, while the entire silicon substrate is 2 × 1.5 cm^2^. A numerical model that accounts for the energy balance of the silicon substrate is used to calculate the average heat flux on the boiling surface. The maximum measurement error on the surface heat flux is estimated at ~5%. A uniform direct current (DC) electric field is generated via a metal grid, laid 6 mm above the boiling surface. The voltage between grid and ground is set at 15 kV, resulting in an average electric field of 2.5 MV/m.

Three different kinds of square pillar microstructures are considered (see Fig. 1), selected based on a theoretical and experimental optimization conducted in saturated pool boiling in normal gravity conditions^19^. Structures are square base pillars of 10 μm height; pillars dimensions are given in Table 1, including the area enhancement. Boiling curves are collected by progressively increasing the surface heat flux (i.e., increasing the Joule heating power) and monitoring the heater temperature. The CHF value coincides with the last point of the boiling curve before a runaway jump of the heater temperature.

**Table 1:** Summary of micropillars geometrical properties.

Surface no.	Pillar side length *L* (μm)	Pillar spacing *D* (μm)	Pillar height *H* (μm)	Ratio between the areas of microstructured and plain surfaces
I	5	10	10	1.8
II	10	15	10	1.5
III	5	20	10	1.3

### Experimental results

**Fig. 3:**
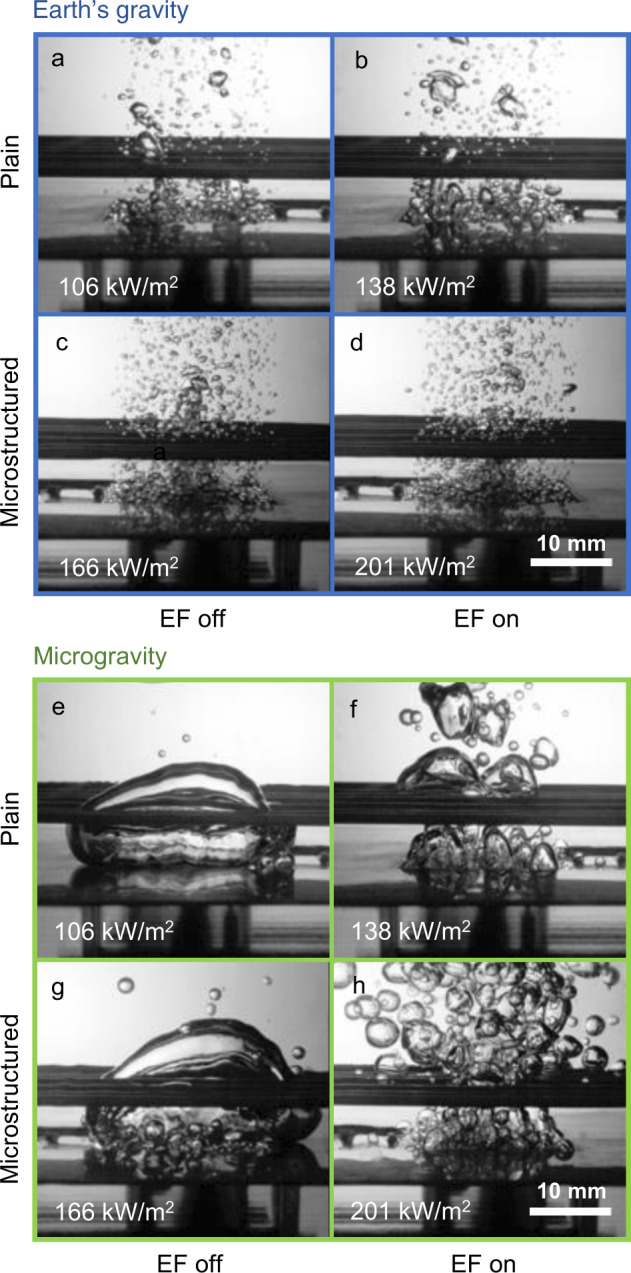
Experimental images of the boiling process in all the tested conditions. On earth (**a**–**d**) and in microgravity conditions (**e**–**h**), on plain (**a**, **b**, **e**, **f**) and microstructured (**c**, **d**, **g**, **h**) surfaces (surface I), with (**a**, **c**, **e**, **g**) and without (**b**, **d**, **f**, **h**) the presence of the electric field. The complete videos are available in Supplementary Movie 1.

Figure 3 shows high-speed video images of the boiling process in all the considered operating conditions. The effect of the electric field is visible in microgravity conditions (figures e–h, green frame). Without the electric field (EF off), bubbles nucleating and growing on the heated surface merge with a large bubble that hovers over it and detaches periodically. This behavior is much different from what we can observe for normal gravity (figures a–d, blue frame). On Earth, bubbles are smaller and move away from the heated surface as soon as they detach. The activation of the electric field on Earth does not affect much the boiling dynamics (no matter the surface). Instead, the effect of the electric field is significant in microgravity conditions. It destroys or suppresses the formation of the large vapor bubble, leading to a boiling process that is qualitatively similar to the one observed in normal gravity. In other words, the body force generated by the electric field is weak compared to buoyancy and does not affect the overall boiling pattern in normal gravity conditions; this agrees with the analysis reported in the Supplementary results. In microgravity instead, the stresses created by the electric field are capable of breaking the liquid–vapor interface, producing smaller bubbles^5,20^. The electric field recreates in microgravity conditions the effects produced in normal gravity by buoyancy forces. Di Marco et al.^21^ have discussed that the triggering mechanism of the transition from nucleate to film boiling may differ from normal to microgravity conditions, leading to a non-continuous trend of CHF values with gravity^22^. The observed boiling dynamics confirms that the electric field may restore, for microgravity conditions, the same mechanisms of normal gravity, as also suggested in previous works^23^.

The effect of microstructures on the boiling dynamics is less flagrant than the electric field. However, in normal gravity conditions, microstructures seem to reduce the bubble size and increase the bubble number. This observation is consistent with the increase of capillary forces promoting the detachment of smaller bubbles from the heated surface. A similar effect is visible in microgravity. Microstructures seem to reduce the size of the bubbles when the electric field is on. Also, microstructures increase the number of small bubbles under the large hovering vapor patch when the electric field is off.

**Fig. 4:**
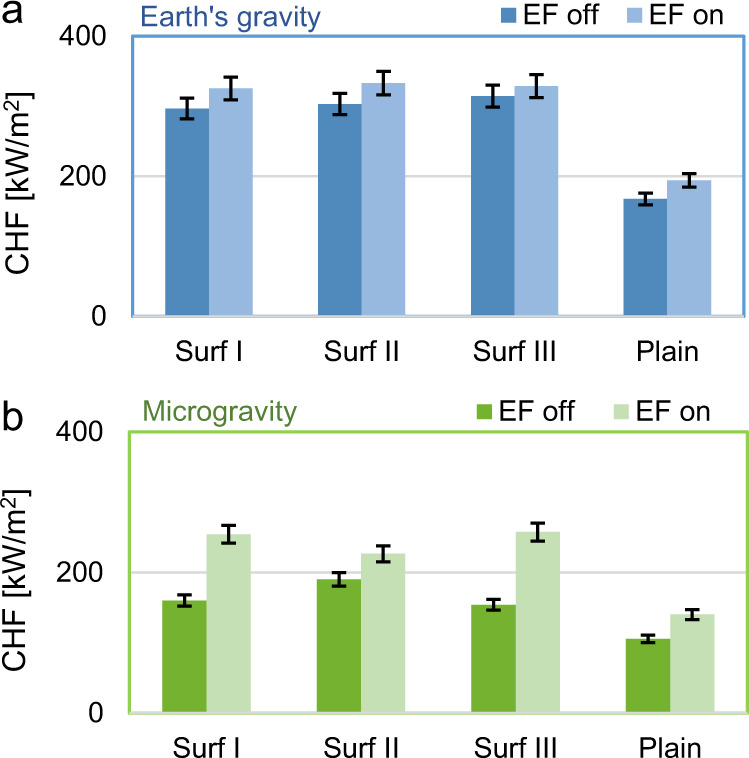
Critical heat flux measured for the tested conditions. **a** Earth conditions: CHF for the four types of surfaces with and without electric field. **b** Microgravity conditions: CHF for the four types of surfaces with and without electric field. Error bars represent the measurement uncertainty (see “Methods” section).

These effects are consistent with the measured CHF values, as summarized in Fig. 4. In Earth’s gravity conditions (top figure), the CHF limit measured on plain surfaces without electric field (i.e., 168 kW/m^2^) agrees with the value predicted by the Zuber^24^ correlation corrected to account for subcooling effects as proposed by Ivey and Morris and adapted to FC-72 by Mudawar and Anderson^25^ (i.e., ~173 kW/m^2^). As shown in Fig. 4a, micropillars increase the CHF limit considerably. However, the activation of the electric field does not have a significant effect (i.e., the CHF enhancement is low, whatever the boiling surface). CHF in microgravity is, as expected, considerably lower than on Earth (see Fig. 4b), around 106 kW/m^2^. Microstructures can increase it, and the benefits of the electric field alone are more pronounced than in Earth’s gravity conditions. However, the most significant CHF enhancement is obtained when the electric field is activated with the microstructured surfaces.

## Discussion

**Fig. 5:**
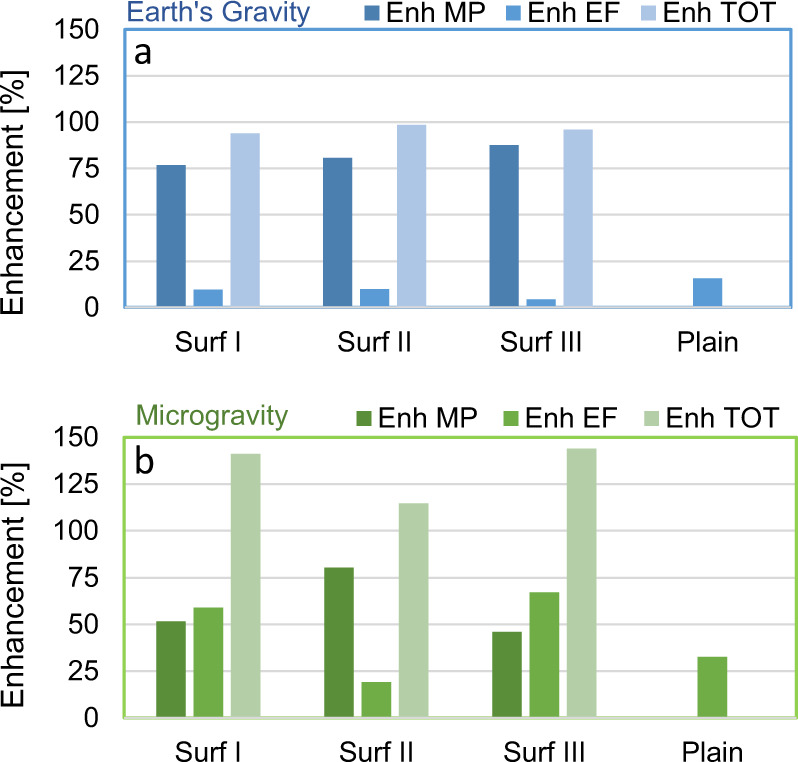
Critical heat flux enhancement achieved for the tested conditions. CHF enhancement with microstructures, electric field, and the combination of both in: **a** Earth conditions. **b** Microgravity conditions.

The CHF enhancement due to micropillars ($\mathrm{E}_{{{{\mathrm{MP}}}}}$), activation of the electric field (${{\mathrm{E}}}_{{{{\mathrm{EF}}}}}$) and both them (${{\mathrm{E}}}_{{{{\mathrm{TOT}}}}}$) can be quantified by the following parameters:

1 $${{\mathrm{E}}}_{{{{\mathrm{MP}}}}} = \frac{{{{\mathrm{q}}}_{{{\mathrm{\mu }}}}^{\prime\prime} - {{\mathrm{q}}}_{{{\mathrm{p}}}}^{\prime\prime} }}{{{{\mathrm{q}}}_{{{\mathrm{p}}}}^{\prime\prime} }}$$

2 $${{\mathrm{E}}}_{{{{\mathrm{EF}}}}} = \frac{{{{\mathrm{q}}}_{{{{\mathrm{EF}}}}}^{\prime\prime} - {{\mathrm{q}}}_{{{{\mathrm{no}}}}\;{{{\mathrm{EF}}}}}^{\prime\prime}}}{{{{\mathrm{q}}}_{{{{\mathrm{no}}}}\;{{{\mathrm{EF}}}}}^{\prime\prime}}}$$

3 $$\mathrm{E}_{{{{\mathrm{TOT}}}}} = \frac{{{{{\mathrm{q}}}}_{{{{\mathrm{\mu }}}} + {{{\mathrm{EF}}}}}^{\prime\prime} - {{{\mathrm{q}}}}_{{{\mathrm{p}}}}^{\prime\prime }}}{{{{{\mathrm{q}}}}_{{{\mathrm{p}}}}^{\prime\prime }}}$$

where ${{\mathrm{q}}}_{{{\mathrm{p}}}}^{\prime\prime}$ is the CHF on the plain surface, ${{\mathrm{q}}}_{{{\mathrm{\mu }}}}^{\prime\prime}$ is the CHF on microstructured surfaces in the absence of electric field, ${{\mathrm{q}}}_{{{{\mathrm{EF}}}}}^{\prime\prime}$ and ${{\mathrm{q}}}_{{{{\mathrm{no}}}}\;{{{\mathrm{EF}}}}}^{\prime\prime}$ are the CHF values with and without the electric field (no matter the surface), and ${{\mathrm{q}}}_{{{{\mathrm{\mu }}}} + {{{\mathrm{EF}}}}}^{\prime\prime}$ is the CHF on microstructured surfaces in the presence of the electric field. The values of these enhancements are summarized in Fig. 5. Note that the total enhancement is not the sum of the other two.

On Earth (Fig. 5a), microstructures enhance CHF of 77–88% compared to the plain surface. In microgravity (Fig. 5b), microstructures alone increase the microgravity CHF of 46–80%, reaching values comparable to the CHF on the plain surface at normal gravity, i.e., these microstructures allow restoring the same boiling performance as on Earth. This finding is meaningful, as it confirms that the capillary effects produced by these micropillars persist even in microgravity conditions. However, the enhancement promoted by microstructures is higher on Earth than in microgravity conditions, i.e., it is tied to the bubble dynamics on the boiling surface. This aspect becomes clearer considering the CHF values in the presence of the electric field. On Earth (Fig. 5a), the effect of the electric field is minimum; this is because buoyancy forces are already effective in removing bubbles. In relative terms, electric field and microstructures (together) enhance the CHF of 96–99%, i.e., only a minor increase compared to microstructures alone. In microgravity conditions (Fig. 5b), instead, the enhancement achieved through the activation of the electric field is much higher. The CHF ranges from 227 to 257 kW/m^2^. The combination of electric field and microstructured enhances the CHF of 114–144%. In other words, the total CHF enhancement is superior to the Earth’s. By breaking the large bubble hovering over the heated surface, the electric field transforms the bubble dynamics (restoring the one observed on Earth). It allows the microstructures to work more efficiently, i.e., there is a synergistic effect.

Ideally, while the electric field is currently limited at 2.5 MV/m by our power supply, we could reach the same CHF as on Earth (with microstructures) by increasing the average electric field. Di Marco and Grassi^26^ showed that this is possible on plain surfaces. Practically, the only limit is the dielectric strength of FC-72 (but there is plenty of room to increase the electric field intensity before that may constitute a problem).

The contribution to CHF enhancement of electric field and microstructures is not the same for all the surfaces in microgravity (Fig. 5b): Surface I shows almost the same enhancement percentage for electric field and microstructures effects, while for Surface II the major contribution is due to microstructures, and for Surface III electric field contribution is slightly higher. Pillar dimensions determine the rewetting capability due to the capillary forces, and determine also local intensifications of electric field, influencing vapor motion and mushroom bubble formation. However, while further investigations are required to elucidate the mechanisms that cause these variations, the effectiveness of microstructures and their synergic action with electric field to enhance the CHF in microgravity has always been observed with all the tested surfaces.

In conclusion, we demonstrate the possibility of enhancing the CHF in microgravity conditions by combining microstructured surfaces and electric fields. Microstructured surfaces improve the capillary forces that facilitate the detachment of small bubbles from the heated surface, whereas the electric field generates a body force that moves bubbles away, mimicking the effect of gravity. Importantly, these two effects are synergistic, i.e., they improve each other. Microstructures alone increase the microgravity CHF of 46–80% compared to a plain surface, reaching values close to the CHF on Earth. When a uniform average electric field of 2.5 MV/m is applied, the boiling patterns are drastically transformed, and the CHF is further increased. In relative terms, the combination of electric field and microstructures enhances the CHF of 114–144% in microgravity compared to a plain surface. Overall, the CHF with microstructures in the presence of the electric field ranges from 227 to 257 kW/m^2^, which is not far from the value measured on Earth for the same surfaces. Also, our observations and analysis suggest that, with stronger electric fields, one could even restore the same CHF value as measured on Earth. However, these findings have the potential to foster the design of very compact and efficient two-phase heating and cooling systems for microgravity applications.

## Methods

### Hardware description

**Fig. 6:**
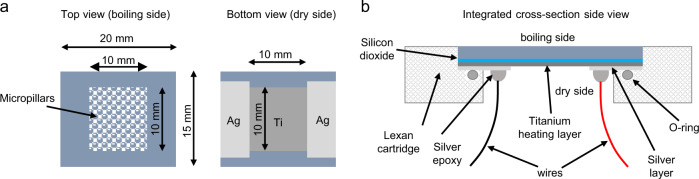
Heated surface schematics (not to scale). **a** Top and bottom view of the silicon surface, with silver and titanium layers for heating. **b** Side view of the surface integrated in the Lexan support; the power wires are glued to the silver layer with conductive silver epoxy.

The heart of the boiling facility consists of the silicon substrates (on which boiling occurs), mounted on a Lexan plate and immersed in FC-72. The silicon substrates (see Fig. 6) are heated by Joule effect by a titanium layer coated on their dry side. Highly conductive silver pads coated on top of the titanium at the sides of the substrate define the active heating surface and serve to connect (with highly conductive silver epoxy glue) two electrical wires. The areas of this heating surface and the structured surface on the boiling side are both 10 × 10 mm^2^. The entire silicon substrate is instead 20 × 15 mm^2^.

**Fig. 7:**
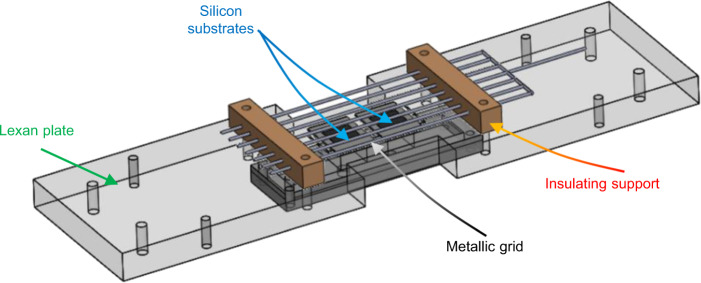
3D CAD model of the test section. The silicon substrates are fixed on a Lexan plate that is kept in the middle of the test cell filled with liquid FC-72. The steel grid is placed above the boiling region with supports of insulating material.

A PT100 sensor (not shown in the picture) is attached at the center of the bottom side of the silicon substrate (i.e., on top of the titanium layer); measurement error of the sensor is ±0.55 °C. Previous IR measures confirmed by numerical simulations showed that the bottom side of the silicon is isothermal during boiling. A numerical model that accounts for the energy balance of the silicon substrate is used to calculate the average heat flux transferred through the 10 × 10 mm^2^ boiling surface using as input the measured electric power transferred to the titanium heating layer and the temperature of the dry side of the silicon substrate, measured by the PT100 sensor. Two heaters are mounted in parallel (see Fig. 7) and are operated one at a time. This is to test two different surfaces during one single flight.

An electrostatic field can be established over the surface by means of a metallic grid laid at 6 mm from the boiling surfaces, which allows vapor to escape (see Fig. 7). A voltage up to 15 ± 0.1 kV DC can be applied between the grid and the ground, connected to a dedicated high-voltage power supply. Numerical simulations showed that in the boiling region (far enough from the grid) the electric field is uniform.

The test section is installed inside a cell of 2 l volume. A high-speed and high-definition optical camera (Ximea MQQ022MG-CM) is used for side-view observations of the boiling process through a Lexan window. A LED illuminating light is mounted on the other side for backlit shadowgraphy. A bellow, operated by two electro-valves, is used to control the cell pressure. The cell temperature is controlled by a cooling/heating loop and by external resistive pad heaters attached to the cell walls. The list of parameters acquired during the operation of the apparatus includes: voltage across and current through the titanium heating layer (used to measure the Joule heating power); silicon substrate bottom temperature; fluid pressure and temperature; environment pressure and temperature; acceleration; video images.

The test facility is mounted on a 2000 × 560 × 580 mm experimental rack, composed by Rose–Krieger aluminum profiles, which satisfies all requirements for parabolic flights. The total mass of the test apparatus is 180 kg.

### Data analysis and measurement uncertainties

**Fig. 8:**
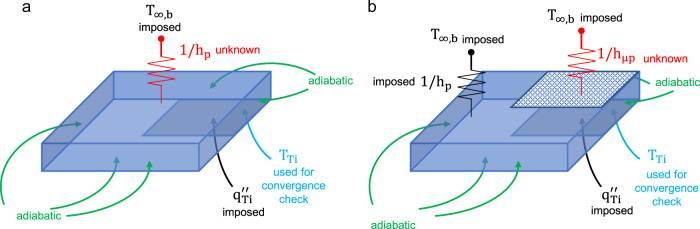
Geometry (not to scale) and boundary conditions for the 3D conduction inverse problem. **a** Plain surface. **b** Engineered surface with microstructures.

Measurement errors are evaluated according to the error propagation theory. Thermal power is calculated as ${P} = {V} \cdot {I}$, where *P* is the power, *V* is the voltage drop measured at the wires ends, *I* is the current passing through the wires. Voltage is directly measured by the acquisition system and current is measured via a shunt resistor. Errors for voltage and current are 0.14% and 1.4%, respectively. Geometrical errors of silicon surface and wiring connections are estimated to be 1%. Based on these values, the error on heat flux measurement can be estimated as 5 kW/m^2^ (i.e., ~1.7%). In order to calculate the heat flux transferred through the 10 × 10 mm^2^ engineered surface in the middle of the surface in contact with the fluid, a MATLAB finite volume model has been developed to solve the 3D conduction problem in the silicon substrate. In order to save on computational costs, only a quarter of the plate is considered. The 3D conduction model uses as input the temperature measured at the bottom side of the silicon and the measured heating power, and adopts the following boundary conditions (see Fig. 8):

- Adiabatic boundary for lateral sides of the plate and bottom surface, except for the titanium layer.
- Imposed flux on the titanium layer (as measured).
- The heat transfer coefficient on the boiling surface (let’s assume a plain surface for now, i.e., Fig. 8a), which is the unknown of the problem (that’s the reason why this problem is inverse), and the liquid temperature (in the bulk).

The problem is solved following the steps listed hereafter:

1. Starting from a guess value of the boiling heat transfer coefficient on the boiling area, we calculate the 3D temperature distribution inside the silicon substrate using the 3D conduction model.
2. If the calculated average bottom surface temperature does not match the measure value, the heat transfer coefficient is updated. This step is repeated until the difference between the measured and calculated temperature is smaller than 0.001 °C. One may note that this value is beyond the precision of the PT100 sensor. However, such stringent convergence criterion is used to guarantee the stability of the inverse problem solution.
3. When the process has converged, we know the boiling heat transfer coefficient and the boiling surface temperature, and thus we can calculate the surface heat flux.

On engineered surface the boundary condition for the substrate side in contact with the fluid are slightly more complicated (Fig. 8b). Since there is a portion of the surface that is engineered and a portion which is plain, the boiling process (and the associated heat transfer coefficient) may be different. In this case, we assign the heat transfer coefficient on the plain portion of the surface. Precisely, we use the mean between 0 and the maximum value measured during the boiling tests on the plain surfaces (i.e., with no pillars at all). Instead, the heat transfer coefficient on the micropillars region is solved iteratively, as discussed before. However, we have confirmed by a sensitive analysis that the choice of lateral heat transfer coefficient (i.e., on the plain portion) affects the heat flux on the engineered surface by less than 5%. In conclusion, the maximum error on the heat flux measurement is estimated at 5%.

### Heater and microstructures fabrication process

The silicon substrate (15 × 20 mm^2^, 0.675 mm thick) is coated with a thin titanium layer on the bottom side. This titanium layer acts as a Joule heater. The electric current does not flow through the silicon substrate because of an electrically insulating silicon dioxide layer that forms spontaneously on the silicon wafer surface before this one is coated with titanium. The titanium layer resistance may vary from heater to heater (in the range from 4 to 6 Ohm) and slightly increases with temperature. Electrical connections are ensured by silver pads that limit the active titanium heating area to 10 × 10 mm^2^.

Microstructures are etched in the central part of the top surface using Deep Reactive Ion Etching (DRIE) ion beam manufacturing. The entire process is described hereafter:

1. Piranha cleaning. The substrate is cleaned in a bath of sulfuric acid (H_2_SO_4_) and hydrogen peroxide (H_2_O_2_) with a 4:1 ratio (note that the mixing of this solution is extremely exothermic). The solution cleans organic compounds and takes between 5 and 10 min.
2. Photolithography. This step serves to create a photoresist mask defining the geometry of the metallic layer to coat (Step 3), e.g., titanium or silver. It must be repeated for each layer and consists of several steps, as listed below:

- Hexamethyldisilazane (HMDS) is applied to the surface of the substrate through vapor priming. It serves as a primer to improve the adhesion of the photoresist.
- The photoresist (AZ5214, typical for image reversal processes) is spin coated on one of the substrate sides in three phases: a dispense phase (6 sec at 500 rpm), a spreading phase (6 s at 750 rpm) and a spin phase (30 s at 3000 rpm).
- The substrate is soft-baked (30 min at 90 °C).
- The desired mask is centered and aligned on top of the substrate.
- The substrate is exposed to UV light for 1.5 s.
- The substrate is soft-baked for 2 min at 120 °C. This step is necessary for image reversal.
- A second exposure (flood, without mask for 90 s) is performed.
- The substrate is developed in a bath of AZ422 for 90 s.

3. E-beam evaporation. The substrates with the photoresist mask created at Step 2 are coated with titanium or silver. This process takes place in an e-beam evaporator. The process takes about 4 h:1.5 h to pump the chamber down to 5.3 × 10^−4^ Pa, 1.5 h for the metal deposition, and 1 h to pump the chamber back to room pressure. After every evaporation, 24 h acetone bath is necessary to remove the photoresist.
4. Photolithography for DRIE. This procedure is used to etch pillars on the silicon substrate.

- HMDS is applied to the surface of the substrate through vapor priming. It serves as a primer to improve the adhesion of the photoresist.
- The photoresist (SPR700, positive photoresist) is spin coated on one of the substrate sides (not coated with metals) in three phases: a dispense phase (6 s at 500 rpm), a spreading phase (4 s at 750 rpm) and a spin phase (30 s at 3500 rpm). These settings are optimized to achieve a photoresist thickness between 1 μm and 1.5 μm, which is necessary to guarantee that the silicon wafer will not be attached by the DRIE process, unless the photoresists has been removed (see below).
- The substrate is soft-baked for 60 s at 95 °C.
- The desired mask is centered and aligned on top of the substrate.
- The wafer is UV exposed for 0.9–1.2 s.
- The substrate is soft-baked for 60 s at 115 °C.
- The substrate is developed in a bath of CD26 for 50 s.

5. DRIE. We use the Bosch process, which consists in alternating an etching stage where SF_6_ plasma is released and attacks silicon, and a passive layer step C_4_F_8_.
6. Die saw cut. The silicon substrate is cut to the desire size using a die saw. This is a very precise tool with a 30-μm-thick blade rotating at around 5000 rpm. It cuts throughout the silicon substrate, up to half of the tape where the substrate is attached. Acetone and UV light are used for a final cleaning of the surface.

### Experimental procedure

**Fig. 9:**
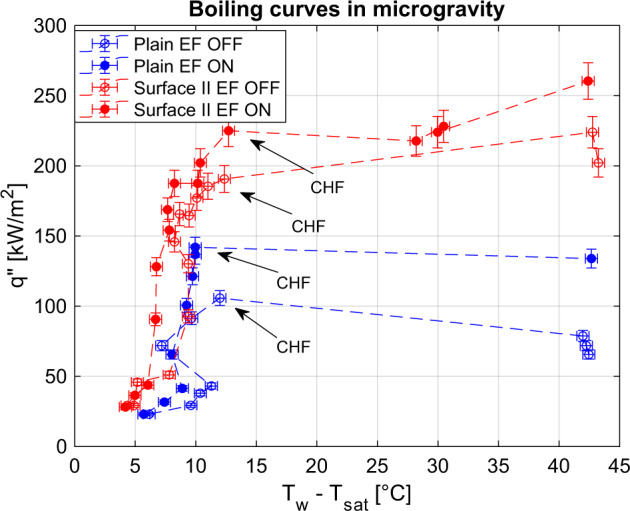
Boiling curves of the plain surface and surface II (example of microstructured surfaces) obtained in microgravity conditions. Error bars represent the measurement uncertainty (see “Methods” section). The abrupt increase of the wall temperature indicates that CHF has been passed.

ESA 71st Parabolic Flight Campaign took place in Bordeaux in May 13–24, 2019. It consisted of 3 days and 31 parabolas each day. In one day, we tested up to two different surfaces. The microgravity duration for each parabola is about 22 s. Experimental temperature and pressure in the boiling cell were reached before the beginning of the parabolas and continuously monitored. The heat flux was regulated before the beginning of each parabola; heat fluxes range from 20 to 300 kW/m^2^ (depending on CHF), with steps of 10–40 kW/m^2^. When microgravity was reached, boiling was kept for almost 11 s without electric field; then, the electric field was switched on and kept for the remaining 11 s. That amount of time was enough to reach thermal stationary conditions (confirmed by numerical calculations and experimental data) and for the effect of gravity transient to be negligible. Each parabola corresponded to a point of the boiling curve; however, we focused mostly on the high heat fluxes (close to CHF), slightly increasing the heat flux between consecutive parabolas in order to find the CHF as accurately as possible. When the CHF limit was exceeded, the temperature of the heater increased abruptly and the power supply was switched off automatically. Figure 9 shows some of the boiling curves obtained on the parabolic flight; the points representing the CHF are marked.

### Reporting summary

Further information on research design is available in the Nature Research Reporting Summary linked to this article.

## Supplementary information

Supplementary Movie 1

Supplementary Information

Reporting Summary

## Data Availability

The data that support the findings of this study are available from the corresponding author upon reasonable request.

## Code availability

The code used in this study and described in detail in Supplementary Material is available from the corresponding author upon reasonable request.
