# Supplementary material for: Critical heat flux enhancement in microgravity conditions coupling microstructured surfaces and electrostatic field
Source: NPJ Microgravity. 2021 Oct 8;7:37. doi: 10.1038/s41526-021-00167-3 (PMC8501093; doi:10.1038/s41526-021-00167-3)
Supplement: Supplementary file 2 — Supplementary Information [file 41526_2021_167_MOESM2_ESM.pdf]

Supplementary Movie 1

The movie shows four video sequences of boiling in microgravity, referring to Fig.3; the sequences cover almost all the microgravity time allowed by a single parabola (transitions are excluded). At second 0, the plain surface without electric field is shown; the measured heat flux is 106 W/m^2. At second 18, the plain surface with the electric field is shown; the measured heat flux is 138 W/m^2. At second 35, the microstructured surface I without electric field is shown; the measured heat flux is 166 W/m^2. At second 51, the microstructured surface I with the electric field is shown; the measured heat flux is 201 W/m^2.

Supplementary results: buoyancy and electric force comparison

When a dielectric liquid boils in an electric field, electric forces rise, especially at liquid-vapor interfaces. Such forces contribute to bubble detachment, vapor movement and interfaces stability together with all the other forces, e.g., buoyancy, surface tension, overpressure, viscous, and inertia forces (to cite the most important).

As Di Marco and Grasssi[1] discussed, different dimensionless numbers can be used to compare the relevance of two or more forces in the general balance of a vapor bubble. We are interested in comparing the ratio between buoyancy force and electric force.

Consider a spherical vapor bubble immersed in an electric field; to estimate the electric force, the dipole approximation can be used[2]:

$$F_E = \frac{2}{3}\pi R_B^2 \frac{(\varepsilon_v - \varepsilon_l)}{(\varepsilon_v + 2\varepsilon_l)} \varepsilon_0 \varepsilon_v \nabla E^2 \tag{1}$$

where ε_v and ε_l are the vapour and liquid permittivity, R_B is the bubble radius and ∇E^2 is the gradient of the square electric field. Electric force F_E and buoyancy force F_b can be compared defining the following dimensionless number:

$$G_{b,E} = \frac{|F_E|}{|F_b|} = \frac{3(\varepsilon_g - \varepsilon_l)}{2(\varepsilon_g + 2\varepsilon_l)} \frac{\varepsilon_0 \varepsilon_g}{(\rho_l - \rho_g)g} \nabla E^2 \tag{2}$$

where ρ_v and ρ_l are the vapour and liquid density and g is the acceleration of gravity.

Even if the electric field distribution of the unperturbed system is uniform (no gradients), local non-uniformities rise in the presence of bubbles. The accurate evaluation of the local non-uniformity can be performed solving the Maxwell's equations knowing the bubbles size and shape. However, a good approximation of the gradients can be obtained considering the mean values of the physical quantities involved, as shown by Saccone et al.[3]. We can approximate ∇E^2 with E^2/h, where h is 6 mm and $E = V/h$ = 15000 V/ 0.006 m = 2.5 MV/m:

$$G_{b,E} = \frac{|F_E|}{|F_b|} = \frac{3(\varepsilon_g - \varepsilon_l)}{2(\varepsilon_g + 2\varepsilon_l)} \frac{\varepsilon_0 \varepsilon_g}{(\rho_l - \rho_g)g} \frac{E^2}{h} \tag{3}$$

Calculating this number for the experiments on ground and considering the properties of FC-72 we obtain $|F_E| = 0.26 \cdot |F_b|$. So, the electric force is present but weaker than buoyancy in normal gravity, as confirmed by experiments. In microgravity however, the value of g is very small ($10^{-2}g$) and the electric force prevails.

Supplementary references

1. Di Marco, P. & Grassi, W. Motivation and results of a long-term research on pool boiling heat transfer in low gravity. *Int. J. Therm. Sci.* **41**, 567–585 (2002).
2. Pethig, R. Dielectrophoresis: Status of the theory, technology, and applications. *Biomicrofluidics* **4**, 1–35 (2010).
3. Saccone, G., Garivalis, A. I. & Di Marco, P. Electrohydrodynamics and boiling: Experiments, numerical calculation and modeling of Maxwell stress tensor and electric force acting on bubbles. *J. Electrostat.* **103**, 103413 (2020).
