# Supplementary material for: Critical heat flux enhancement in microgravity conditions coupling microstructured surfaces and electrostatic field
Source: NPJ Microgravity. 2021 Oct 8;7:37. doi: 10.1038/s41526-021-00167-3 (PMC8501093; doi:10.1038/s41526-021-00167-3)
Supplement: Supplementary file 3 — Reporting Summary [file 41526_2021_167_MOESM3_ESM.pdf]

nature portfolio

Corresponding author(s): Paolo di Marco

Last updated by author(s): Jul 27, 2021

Reporting Summary

Nature Portfolio wishes to improve the reproducibility of the work that we publish. This form provides structure for consistency and transparency in reporting. For further information on Nature Portfolio policies, see our Editorial Policies and the Editorial Policy Checklist.

Statistics

For all statistical analyses, confirm that the following items are present in the figure legend, table legend, main text, or Methods section.

n/a	Confirmed	
☒	☐	The exact sample size (*n*) for each experimental group/condition, given as a discrete number and unit of measurement
☒	☐	A statement on whether measurements were taken from distinct samples or whether the same sample was measured repeatedly
☒	☐	The statistical test(s) used AND whether they are one- or two-sided<br>*Only common tests should be described solely by name; describe more complex techniques in the Methods section.*
☒	☐	A description of all covariates tested
☒	☐	A description of any assumptions or corrections, such as tests of normality and adjustment for multiple comparisons
☒	☐	A full description of the statistical parameters including central tendency (e.g. means) or other basic estimates (e.g. regression coefficient) AND variation (e.g. standard deviation) or associated estimates of uncertainty (e.g. confidence intervals)
☒	☐	For null hypothesis testing, the test statistic (e.g. *F*, *t*, *r*) with confidence intervals, effect sizes, degrees of freedom and *P* value noted<br>*Give P values as exact values whenever suitable.*
☒	☐	For Bayesian analysis, information on the choice of priors and Markov chain Monte Carlo settings
☒	☐	For hierarchical and complex designs, identification of the appropriate level for tests and full reporting of outcomes
☒	☐	Estimates of effect sizes (e.g. Cohen's *d*, Pearson's *r*), indicating how they were calculated

Our web collection on statistics for biologists contains articles on many of the points above.

Software and code

Policy information about availability of computer code

Data collection	NI Labview 2016
Data analysis	Matlab 2016a

For manuscripts utilizing custom algorithms or software that are central to the research but not yet described in published literature, software must be made available to editors and reviewers. We strongly encourage code deposition in a community repository (e.g. GitHub). See the Nature Portfolio guidelines for submitting code & software for further information.

Data

Policy information about availability of data

All manuscripts must include a data availability statement. This statement should provide the following information, where applicable:

- Accession codes, unique identifiers, or web links for publicly available datasets
- A description of any restrictions on data availability
- For clinical datasets or third party data, please ensure that the statement adheres to our policy

The data that support the findings of this study are available from the corresponding author upon reasonable request.

Field-specific reporting

Please select the one below that is the best fit for your research. If you are not sure, read the appropriate sections before making your selection.

☐ Life sciences ☐ Behavioural & social sciences ☒ Ecological, evolutionary & environmental sciences

For a reference copy of the document with all sections, see nature.com/documents/nr-reporting-summary-flat.pdf

Ecological, evolutionary & environmental sciences study design

All studies must disclose on these points even when the disclosure is negative.

Study description	We run pool boiling experiments with a dielectric fluid (FC-72) on Earth and on board an ESA parabolic flight aircraft able to cancel the effects of gravity, testing both highly-wetting microstructured surfaces and plain surfaces and applying an external electric field that creates gravity-mimicking body forces. Our results reveal that microstructured surfaces, known to enhance the critical heat flux on Earth, are also useful in microgravity. An enhancement of the microgravity critical heat flux on a plain surface can also be obtained using the electric field. However, the best boiling performance is achieved when these techniques are used together. The effects created by microstructured surfaces and electric fields are synergistic, demonstrating the potential of this synergistic approach towards very compact and efficient two-phase heat transfer systems for microgravity applications.
Research sample	Sample consist of a silicon chip 1 cm x 1 cm x 0.5 mm electrically heated ad where boiling occurs. One chip is smooth while other 3 chips where ion-beam etched to obtain three kinds of microstructures on boiling surface.
Sampling strategy	N/A
Data collection	Data were collected during parabolic flight, by most of the authors (listed in the authors contribution section).
Timing and spatial scale	The parabolic flight campaign is three days long. Every flight day 31 parabolas are performed; each parabola is around 1 and a half minutes long (22 seconds of microgravity).
Data exclusions	No data exclusions.
Reproducibility	Due to limited parabolic flight opportunities, only one set of measures was performed.
Randomization	N/A
Blinding	N/A

Did the study involve field work? ☒ Yes ☐ No

Field work, collection and transport

Field conditions	Inside a parabolic flight aircraft AirZEROG, microgravity environment
Location	Novespace Aircraft AirZEROG
Access & import/export	N/A
Disturbance	N/A

Reporting for specific materials, systems and methods

We require information from authors about some types of materials, experimental systems and methods used in many studies. Here, indicate whether each material, system or method listed is relevant to your study. If you are not sure if a list item applies to your research, read the appropriate section before selecting a response.

Materials & experimental systems

n/a	Involved in the study
☒	☐ Antibodies
☒	☐ Eukaryotic cell lines
☒	☐ Palaeontology and archaeology
☒	☐ Animals and other organisms
☒	☐ Human research participants
☒	☐ Clinical data
☒	☐ Dual use research of concern

Methods

n/a	Involved in the study
☒	☐ ChIP-seq
☒	☐ Flow cytometry
☒	☐ MRI-based neuroimaging
